# Intraluminal Bowel Erosion: A Rare Complication of Retained Gallstones after Cholecystectomy

**DOI:** 10.1155/2016/7396981

**Published:** 2016-09-14

**Authors:** Alex B. Blair, Nathaniel McQuay

**Affiliations:** Department of Surgery, Johns Hopkins Bayview Medical Center, Baltimore, MD, USA

## Abstract

Laparoscopic cholecystectomy for acute cholecystitis and cholelithiasis is one of the most common operations performed in the United States. Inadvertent perforation and spillage of gallbladder contents are not uncommon. The potential impact of subsequent retained gallstones is understated. We present the case of an intraperitoneal gallstone retained from a previous cholecystectomy eroding into the bowel and leading to intraluminal mechanical bowel obstruction requiring operative intervention. This case illustrates the potential risks of retained gallstones and reinforces the need to diligently collect any dropped stones at the time of initial operation.

## 1. Introduction

Laparoscopic cholecystectomy for acute cholecystitis and cholelithiasis is one of the most common operations performed in the United States. The operative risks of bile leak and wound infection and the low incidence of common bile duct injury have been well documented. However, the potential impact of retained gallstones is understated. We present a case of an intraperitoneal gallstone retained from a previous cholecystectomy eroding into the bowel leading to intraluminal mechanical bowel obstruction.

## 2. Case Presentation

**Figure 1:**
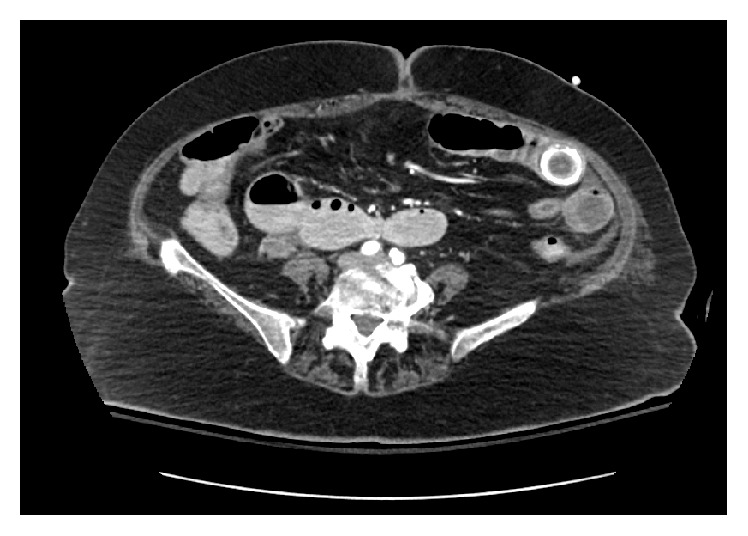
Abdomen and pelvis CT revealing intraluminal gallstone in the left lower quadrant leading to high grade bowel obstruction.

The Acute Care Surgery service was consulted to evaluate a 79-year-old female with new onset fever, abdominal pain, and emesis. Her complex past medical history included atrial fibrillation requiring anticoagulation, hypertension, recent* Clostridium difficile* infection, and remote stroke requiring tracheostomy and percutaneous gastrostomy tube placement. Physical examination of the abdomen was notable for distention and diffuse tenderness to palpation. Plain abdominal film demonstrated a bowel gas pattern concerning an acute intestinal obstruction for which an abdominal computerized tomography scan was performed for further delineation. A 2.7 cm hyperdense lesion suspicious for a stone was appreciated intraluminally within the jejunum causing a high grade bowel obstruction (Figure 1).

**Figure 2:**
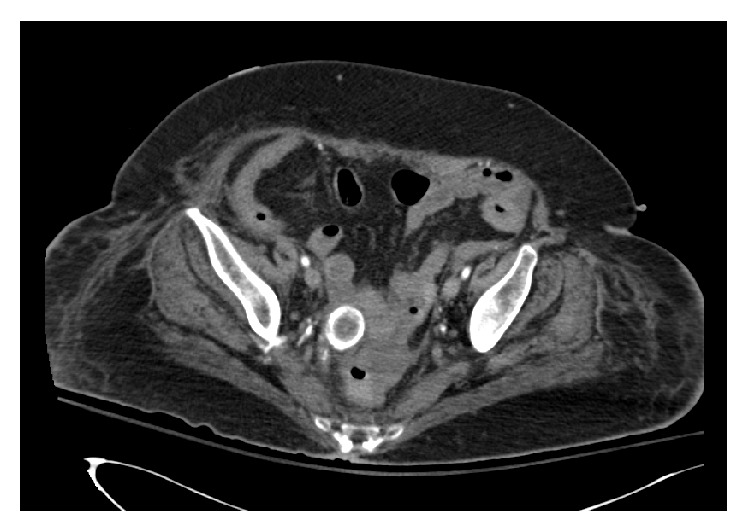
Abdomen and pelvis CT one month prior to obstructive presentation revealing large gallstone resting in the pelvis outside of the bowel lumen.

This patient's surgical history included a laparoscopic cholecystectomy two months previously for gangrenous cholecystitis. The prior operative report revealed that the retrieval bag had ruptured on removal, and multiple gallstones were spilled into the intraperitoneal space. Complete retrieval was unsuccessful and gallstones were left behind. Of note, the patient had received an additional CT scan in the time span between the prior cholecystectomy and her current obstructive presentation due to an infectious colitis. At that time, the 2.7 cm stone was located resting dependently in the pelvis against the jejunum (Figure 2). We hypothesize that this stone was a nidus for a chronic inflammatory reaction, with subsequent erosion into the adjacent bowel and development of gallstone ileus.

The patient was taken to the operating room for planned exploratory laparotomy. Intraoperatively a segment of jejunum was found adherent to the pelvis and 50 cm distally an intraluminal 2.7 cm gallstone. The stone was milked from the bowel and a short jejunal segment was resected. A primary stapled anastomosis was performed. Further exploration revealed no additional stones and absence of any cholecystoenteric fistulae.

## 3. Discussion

Gallstone ileus is an uncommon cause of mechanical intestinal obstruction occurring most frequently in the female geriatric population. Inflammation in the gallbladder bed coupled with the pressure effect of a gallstone leads to cholecystoenteric fistulae allowing passage of stones into the bowel. Larger stones greater than 2 cm are less likely to be successfully passed through the bowel and can become impacted in the distal small intestine thus leading to a mechanical obstruction [1]. Gallstone ileus must be relieved surgically with current recommendations for a two-staged procedure: first an expeditious enterotomy and removal of the stone early to prevent bowel necrosis, followed by fistula repair [1]. This case is unique, as the gallbladder had previously been removed and no biliary-enteric fistulae were appreciated. The presence of gallstone ileus after cholecystectomy is unusual. To the authors' best knowledge there are only three prior reports of a retained gallstone eroding into the small or large bowel with a range of two months to eight years after initial procedure [2–4]. In each of these cases the gallstone of interest measured 3–6 cm in size.

Laparoscopic cholecystectomy is a commonly performed procedure for acute cholecystitis and cholelithiasis. Inadvertent perforation and spillage of gallbladder contents are not uncommon. Although rarely clinically significant, it is prudent to recognize that subsequent retained stones are not benign. Previous reports have documented an approximately 1–12% complication rate from stones left in the intraperitoneal cavity [5]. Complications present in the form of nonspecific abdominal pain, inflammatory reactions, or intra-abdominal abscesses [4, 6]. However, more dramatic presentations of stone erosion into abutting structures including the diaphragm, chest wall, bladder, and small and large bowel have been reported [4, 6, 7]. These complications can present months to years after the initial operation thus leading to a confusing clinical picture as to the underlying cause.

Perforation of the gallbladder most frequently occurs as a result of retraction or during dissection of the gallbladder from the liver bed. An acutely infected, inflamed, or thin walled gallbladder is more easily ruptured and needle decompression should be considered. If rupture does occur, every appropriate effort to recover dropped stones must be made. Previous reviews have recommended against laparotomy for unretrieved gallstones [5]. However, in the scenario of a large >2 cm, intraperitoneal stone that is unable to be successfully recovered laparoscopically, conversion to a small midline laparotomy may be of benefit and should be considered. If a retained gallstone is unable to be recovered, detailed documentation is indicated in order to help efficiently recognize the etiology of potential future complications. Finally in the informed consent process, patients must always be properly educated about complete preoperative risk, including that of retained stones.

## References

[1] Halabi W. J., Kang C. Y., Ketana N. (2014). Surgery for gallstone ileus: a nationwide comparison of trends and outcomes. *Annals of Surgery*.

[2] Draganic B. D., Reece-Smith H. (1997). Gallstone ileus without a gallbladder. *Annals of the Royal College of Surgeons of England*.

[3] Ivanov I., Beuran M., Venter M. D. (2012). Gallstone ileus after laparoscopic cholecystectomy. *Journal of medicine and life*.

[4] Habib E., Elhadad A. (2003). Digestive complications of gallstones lost during laparoscopic cholecystectomy. *HPB*.

[5] Demirbas B. T., Gulluoglu B. M., Aktan A. O. (2015). Retained abdominal gallstones after laparoscopic cholecystectomy: a systematic review. *Surgical Laparoscopy, Endoscopy and Percutaneous Techniques*.

[6] Tumer A. R., Yüksek Y. N., Yasti A. C., Gözalan U., Kama N. A. (2005). Dropped gallstones during laparoscopic cholesystectomy: the consequences. *World Journal of Surgery*.

[7] Gaster R. S., Berger A. J., Ahmadi-Kashani M., Shrager J. B., Lee G. K. (2014). Chronic cutaneous chest wall fistula and gallstone empyema due to retained gallstones. *BMJ Case Reports*.

